# Effects of Phosphorus and Boron Compounds on Thermal Stability and Flame Retardancy Properties of Epoxy Composites

**DOI:** 10.3390/polym14194005

**Published:** 2022-09-24

**Authors:** Corneliu Hamciuc, Tăchiță Vlad-Bubulac, Diana Serbezeanu, Ana-Maria Macsim, Gabriela Lisa, Ion Anghel, Ioana-Emilia Şofran

**Affiliations:** 1 Department of Polycondensation and Thermally Stable Polymers, “Petru Poni” Institute of Macromolecular Chemistry, 41A, Grigore Ghica Voda Alley, 700487 Iasi, Romania; 2 Department of Chemical Engineering, Gheorghe Asachi Technical University of Iasi, Faculty of Chemical Engineering and Environmental Protection, Bd. Mangeron 73, 700050 Iasi, Romania; 3 Police Academy “Alexandru Ioan Cuza”, Fire Officers Faculty, Morarilor Str. 3, Sector 2, 022451 Bucharest, Romania

**Keywords:** epoxy composites, phosphorus-containing flame retardant, boric acid, thermal stability, flame resistant

## Abstract

While plastics are regarded as the most resourceful materials nowadays, ranging from countless utilities including protective or decorating coatings, to adhesives, packaging materials, electronic components, paintings, furniture, insulating composites, foams, building blocks and so on, their critical limitation is their advanced flammability, which in fire incidents can result in dramatic human fatalities and irreversible environmental damage. Herein, epoxy-based composites with improved flame-resistant characteristics have been prepared by incorporating two flame retardant additives into epoxy resin, namely 6-(hydroxy(phenyl)methyl)-6H-dibenzo[c,e][1,2]oxaphosphinine-6-oxide (PFR) and boric acid (H_3_BO_3_). The additional reaction of 9,10-dihydro-oxa-10-phosphophenanthrene-10-oxide (DOPO) to the carbonyl group of benzaldehyde yielded PFR, which was then used to prepare epoxy composites having a phosphorus content ranging from 1.5 to 4 wt%, while the boron content was 2 wt%. The structure, morphology, thermal stability and flammability of resulted epoxy composites were investigated by FTIR spectroscopy, scanning electron microscopy (SEM), thermogravimetric analysis, differential scanning calorimetry, and microscale combustion calorimetry (MCC). Thermogravimetric analysis indicated that the simultaneous incorporation of PFR and H_3_BO_3_ improved the thermal stability of the char residue at high temperatures. The surface morphology of the char residues, studied by SEM measurements, showed improved characteristics in the case of the samples containing both phosphorus and boron atoms. The MCC tests revealed a significant reduction in flammability as well as a significant decrease in heat release capacity for samples containing both PFR and H_3_BO_3_ compared to the neat epoxy thermoset.

## 1. Introduction

Epoxy resins represent one of the most developed classes of thermosetting polymers due to their numerous applications in the construction, transportation, aerospace industry, biomedical systems, electrical and electronic fields. They have excellent mechanical properties, good thermal and chemical resistance, good electrical insulating properties, and low shrinkage after curing [1,2,3]. Epoxy resins have been widely used as polymeric matrices for the preparation of composites with improved characteristics. The properties of composites depend on the structure of the resins, curing agent, and organic or inorganic fillers [4,5,6]. However, cured epoxy resins have high flammability, which reduces their application in areas where flame-resistant polymeric materials are required. A commonly used method to improve their flame resistance characteristics is to incorporate flame retardant additives into epoxy resins [7,8,9]. In the last decades, conventional halogenated flame retardants have been frequently mixed with epoxy resins to improve their flame resistance, but these compounds can release toxic chemicals into the environment and by combustion can produce very dangerous toxic compounds [10,11]. Therefore, much research has been done to develop new environmentally friendly high-efficiency flame retardant additives.

Phosphorus-containing flame retardants have received extensive attention because they are environmentally friendly, highly efficient in reducing the flammability of polymers, and do not produce very toxic compounds by burning [12,13,14,15,16,17,18]. A special class of phosphorus-containing flame retardants frequently used to improve the flame-resistant properties of epoxy resins is represented by 9,10-dihydro-9-oxa-10-phosphaphenanthrene-10-oxide (DOPO) and their derived compounds [19,20,21]. It has been demonstrated in numerous studies that due their high degree of aromaticity they exhibit high flame retardant performance. These flame retardants present a twofold mechanism of flame inhibition: on one hand in the gas phase by producing PO· radicals, and on the other hand in the solid phase by their direct interaction with the decomposition species evolved from the polymer backbone, to produce a more compact carbonaceous char layer on the surface of the polymeric material [8,20,21,22,23,24,25,26].

A literature survey reveals that boron-containing compounds can be also used as flame retardant additives for epoxy resins [27,28]. These compounds improve the flame resistance of the polymers by acting in the gas and the condensed phases. They decompose endothermically, reducing the temperature of the system and producing water that can dilute flammable gases that result from the pyrolysis process [29]. In the condensed phase, they improve the formation and stabilization of a protective char layer on the polymer surface [30,31]. The presence of inorganic particles in the epoxy resin may provide useful characteristics such as heat resistance and flame retardancy. The incorporation of boric acid (H_3_BO_3_) particles into epoxy resin leads to a significant modification of the structure and properties of epoxy thermosets, and to a reduction in the flammability of the composites. H_3_BO_3_ is colorless, inexpensive, and exhibits low volatility and relatively low toxicity. This compound eliminates water at a temperature higher than 100 ᵒC [32,33]. The water released during its decomposition reduces the temperature and can dilute the combustible gases in the combustion zone. At higher temperatures, H_3_BO_3_ is converted to boric trioxide (B_2_O_3_) [34,35] which can form a glassy film on the burning surface of the polymer, thus inhibiting the diffusion of flammable gases in the combustion zone and reducing the flame spread on the polymer surface [36,37].

One way to increase the effectiveness of flame retardants and to reduce the costs associated with their use is to incorporate into epoxy resins two flame retardants that have a synergistic effect in reducing flammability. Several studies have demonstrated that boron-containing flame retardants have a synergistic effect with flame retardants containing phosphorus and nitrogen atoms in improving the flame retardancy of epoxy resins [28]. They improve the char formation with efficient barrier properties and promote the increase in the phosphorus content in the condensed phase [38,39].

In this study, a phosphorus-containing DOPO derivative (PFR) and H_3_BO_3_ were introduced into epoxy resin to improve its flame resistance. The main advantage of choosing PFR and H_3_BO_3_, over other phosphorus-containing flame retardants with complicated architectures that require difficult and time-consuming preparation methods, is related to the simple and cost-effective synthetic procedure of PFR and the ready commercial availability of boric acid, which is doubled by the expected synergistic effect of the two flame retardants. The effect of H_3_BO_3_ and PFR containing phosphorus on decreasing the flammability of epoxy resin was studied. Scanning electron microscopy was used to investigate the morphology of the cured epoxy composites and the char layer resulting from the pyrolysis of the samples. The thermal stability and flame retardance of the composites were investigated by thermogravimetric analysis and microscale combustion calorimetry tests.

## 2. Materials and Methods

### 2.1. Materials

The 9,10-Dihydro-oxa-10-phosphophenanthrene-10-oxide (DOPO, 14.32% P) was purchased from Chemos GmbH (Germany) and was freshly dehydrated before use. Benzaldehyde and H_3_BO_3_ (17.48% B, having the mean particle size distribution of 74.395 μm) were supplied by Sigma-Aldrich and used as received. The two-component based epoxy resin used as the polymeric matrix was supplied by DEVE PRODEXIM Oradea, Romania. The first component (EP) consists in a mixture of bisphenol A diglycidyl ether and an adduct of oxirane with mono (C12-C14-alkoxy) methyl derivatives. The second component (HA), used as a hardener, is a mixture of benzyl alcohol, 3-aminomethyl-3,5,5-trimethylcyclohexylamine, m-phenylenebis(methylamine) and bisphenol-A.

### 2.2. Synthesis of 6-(Hydroxy(phenyl)methyl)-6H-dibenzo[c,e][1,2]oxaphosphinine-6-oxide

The 6-(Hydroxy(phenyl)methyl)-6H-dibenzo[c,e][1,2]oxaphosphinine-6-oxide (PFR) was prepared by the additional reaction of the P-H group of DOPO to the carbonyl group of benzaldehyde, as was previously described in the literature [40]. Benzaldehyde (10.6 g, 0.1 mol), DOPO (23.76 g, 0, 11 mol) and toluene (190 mL) were placed in a 500 mL three-necked glass flask equipped with a magnetic stirrer, temperature regulator, and reflux condenser. The reaction mixture was heated at 110 °C for 10 h under a nitrogen atmosphere. After the reaction was complete, the mixture was cooled to ambient temperature and the resulting precipitate was filtered off. The product was dried in a vacuum oven at 60 °C for 4 h.

Yield: 93%. (30.97% P).

FTIR (KBr, cm^−1^): 935 (P–O–Ph stretching vibrations), 1203 (P=O stretching vibrations), 1474 (P–Ph aromatic ring in-plane stretching vibrations), 3245 (O–H stretching vibrations).

^1^H NMR (CDCl_3_-*d1*): 3.15 (1 H, bs, OH one isomer), 3.58 (1 H, bs, OH second isomer), 5.26 (1 H, d, JH-P = 6.9 Hz, H-5 one isomer), 5.31 (1 H, d, JH-P = 9.4 Hz, H-5-s isomer), 7.19–7.08 (8 H, m, H-1 both isomers, H-2 both isomers, H-14 both isomers, H-16 both isomers), 7.33–7.23 (7 H, m, H-15 both isomers, H-3 both isomers), 7.47–7.40 (2 H, m, H-9 both isomers), 7.72–7.63 (3.5 H, m, H-8 both isomers, H-10 one isomer, H-13 one isomer), 7.97–7.81 (4 H, m, H-7 both isomers, H-10 s isomer, H-13 s isomer).

^13^C NMR (CDCl_3_-*d1*): 73.55 (d, JC-P = 113.1 Hz, C-5 one isomer), 73.73 (d, JC-P = 109.6 Hz, C-5 s isomers), 119.93–119.86 (C-16 both isomers), 121.82–121.10 (m, C-11 both isomers, C-12 both isomers), 122.9 (d, JC-P = 11.1 Hz, C-7 one isomer), 123.3 (d, JC-P = 10.0 Hz, C-7 s isomer), 124.2 (d, JC-P = 7.0 Hz, C-14 both isomers), 124.8 (d, JC-P = 11.8 Hz, C-13 both isomers), 127.02–126.97 (m, C-3 both isomers), 128.31–127.91 (m, C-9 both isomers, C-1 both isomers, C-2 both isomers), 130.4 (d, JC-P = 8.3 Hz, C-15 both isomers), 132.10–131.88 (m, C-10 both isomers), 133.7 (d, JC-P = 10.2 Hz, C-8 both isomers), 134.9 (d, JC-P = 15.9 Hz, C-4 both isomers), 136.92–136.79 (m, C-6 both isomers), 150.35–150.18 (m, C-17 both isomers).

### 2.3. Preparation of Epoxy Resin Composites

Various amounts of PFR were mixed with Epoxy, under stirring at 130 °C for 30 min. The mixtures were cooled to 50 °C, and H_3_BO_3_ was introduced. They were ultrasonicated for 30 min and cooled to 25 °C. Then HA hardener was added, and the stirring was continued for 30 min. The product was cured at room temperature for 24 h, and at 60 °C for 4 h. The formulation of the epoxy resin, PFR and H_3_BO_3_ pre-curing mixtures is listed in Table 1.

### 2.4. Measurements

The structure of PFR was investigated by using FTIR and NMR spectroscopy. The FTIR spectrum was recorded on a FTIR Bruker Vertex 70 Spectrophotometer. The proton and carbon NMR experiments were recorded on Bruker Avance NEO 400 MHz operating at 400.1 and 100.6 MHz, equipped with a 5 mm direct detection four nuclei probe (H, C, Si, F). The phosphorus NMR spectrum was recorded on a Bruker Avance NEO 600 MHz operating at 600.1 MHz and 242.9 MHz, equipped with a 5 mm inverse detection multinuclear probe. ^1^H and ^13^C NMR chemical shifts (δ) in ppm are calibrated to residual solvent peaks (CDCl_3_, 7.26 ppm for ^1^H and 77.01 ppm for ^13^C).

The structure of the thermosets was determined by FTIR spectroscopy using a BioRad ‘FTS 135′ FTIR spectrometer equipped with a Specac “Golden Gate” ATR accessory. A LUMOS Microscope Fourier Transform Infrared (FTIR) spectrophotometer (Bruker Optik GmbH, Ettlingen, Germany), equipped with an attenuated total reflection (ATR) device, was used to record the scans between 4000 and 600 cm^−1^ at a resolution of 4 cm^−1^.

Microscopic investigations of epoxy thermosets and of their corresponding chars were performed on an Environmental Scanning Electron Microscope Type Quanta 200, operating at 10 kV with secondary electrons in a low vacuum mode (LFD detector). The Quanta 200 microscope is equipped with an Energy Dispersive X-Ray (EDX) system for qualitative and quantitative analysis and elemental mapping.

Thermogravimetric (TG) curves and thermogravimetric derivative (DTG) curves of PFR and epoxy thermosets were recorded with Mettler Toledo TGA-SDTA851e equipment, in a nitrogen atmosphere, and a heating rate of 10 °C min^−1^, in the temperature range of 25–800 °C. Differential scanning calorimetry (DSC) measurements of PFR and epoxy thermosets were carried out using a Mettler Toledo DSC1 type device in an inert atmosphere, with a heating rate of 10 °C min^−1^ and nitrogen purge at 100 mL min^−1^.

The flammability behavior of PFR and epoxy thermosets was tested using an FTT Micro Calorimeter [41].

## 3. Results and Discussion

### 3.1. Synthesis and Characterization of PFR

**Figure 1:**
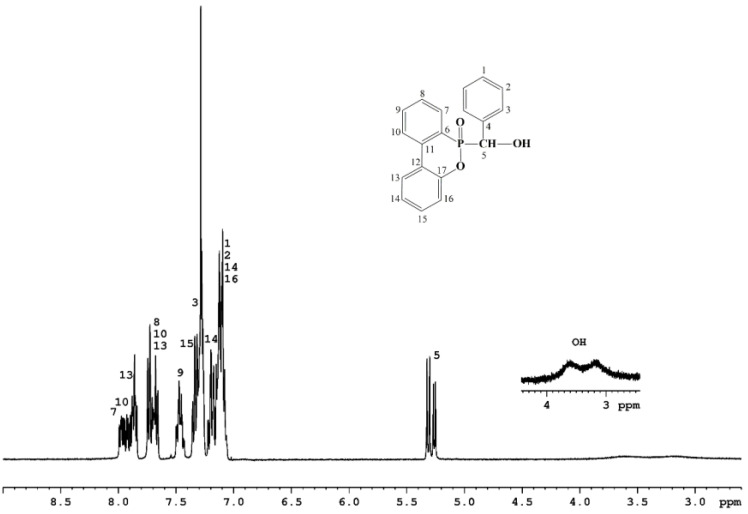
^1^H NMR spectrum of PFR.

PFR was prepared by adding DOPO monomer to the carbonyl group of benzaldehyde, following an adapted method previously reported [40]. The reaction took place in toluene, at 110 °C for 10 h. The structure of PFR was characterized by FTIR, ^1^H NMR, ^13^C NMR and ^31^P NMR spectroscopy. The NMR analysis of PFR showed the existence of two diastereomers in molar ratio of 1:0.7. This phenomenon has been reported in the literature [42] and it was attributed to the chirality of the phosphorus stereocenter of DOPO. The presence of the phosphorus atom in the structure complicates the shape of the signals in the NMR spectra. The ^1^H NMR spectrum of PFR displays some characteristic signals: the hydroxyl group has a broad resonance signal in the region 3.15–3.58 ppm, the CH group proton was associated with the two doublets (one for each isomer) from 5.31 ppm and 5.26 ppm, and all aromatic protons have complex resonance signals were in the region 7.00–8.00 ppm, mainly due to the proton–phosphorus couplings, and also due to the presence of the two isomers causing more overlap of the signals (Figure 1).

**Figure 2:**
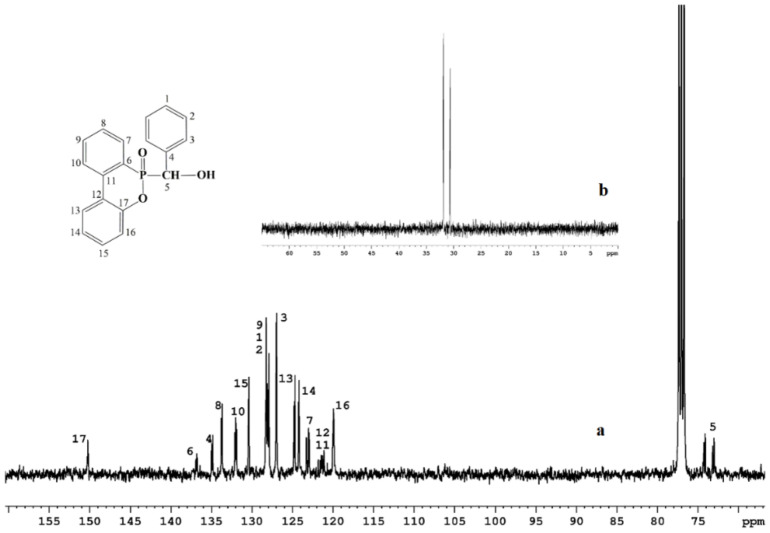
^13^C NMR (**a**) and ^31^P NMR (**b**) spectra of PFR.

In the case of the carbon spectrum, all the signals appear as doublets or multiplets due to carbon–phosphorus coupling. As can be seen in Figure 2, the CH group has two characteristic doublets at 73.5 ppm and 73.7 ppm while the signals for the aromatic carbon atoms appear in the range 118 ppm to 151 ppm, the quaternary carbon directly linked to oxygen being the most de-shielded in the interval 150.1–150.3 ppm. The existence of the two isomers is clearly seen in the ^31^P NMR spectra, by the presence of two signals at 31.88 ppm and 30.65 ppm, respectively (Figure 2 inset).

**Figure 3:**
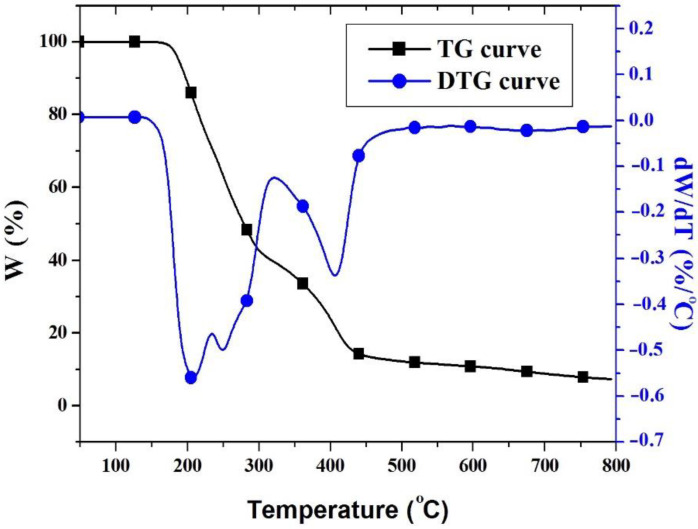
TG and DTG curves of PFR.

The thermal properties of PFR were investigated by TGA analysis. The TG and DTG curves of PFR showed that it decomposed in two steps (Figure 3). The first decomposition step was in the temperature range of 180–320 °C, which may be attributed to the catalysis of acidic phosphorus-based products evolved during the thermal decomposition of oxaphosphinine-6-oxide structure, while the second step took place in the interval of 320–520 °C as a result of the further thermal decomposition of the enriched aromatic carbonaceous structure [43]. The maximum weight loss rate in the first step of decomposition was 0.55 %/°C while the maximum weight loss rate of the second step of decomposition was lower (0.34 %/°C). The temperature at which the mass loss rate is the highest in the thermal decomposition process of the first step of decomposition was 208 °C, while that of the second step of decomposition appeared at 408 °C.

### 3.2. Structural and Morphological Characterization of Epoxy-Based Composites

**Figure 4:**
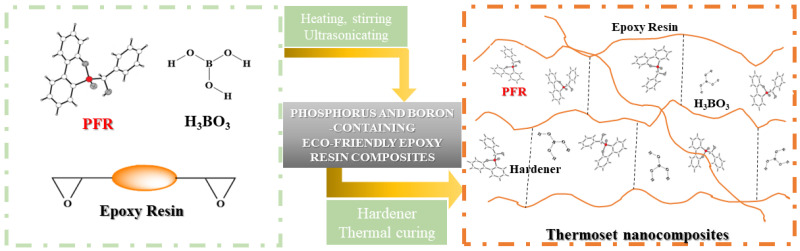
Graphical representation of epoxy composites synthetic pathway.

Epoxy-based composites were prepared by incorporation of PFR and H_3_BO_3_ in epoxy resin (Figure 4). The PFR content was adjusted so that the phosphorus atom concentration varies between 0 and 4 wt%. H_3_BO_3_ content was calculated to have a concentration of 2 wt% of boron in the thermosets (Table 1).

**Figure 5:**
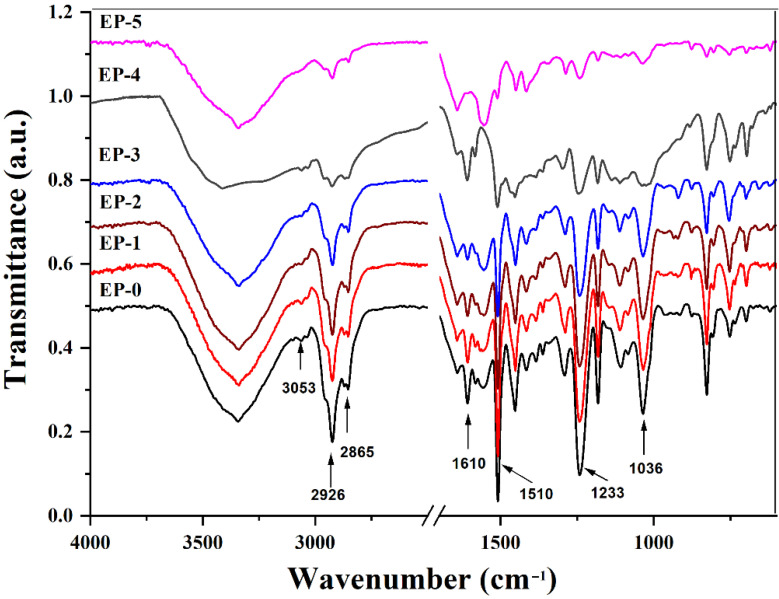
FTIR spectra of EP epoxy composites.

The resulting thermoset structures were investigated by FTIR spectroscopy (Figure 5). The neat EP-0 system presented characteristic absorption bands at 3350 cm^−1^ (O-H stretching vibrations), 3053 cm^−1^ (C–H tension of the methylene group of the epoxy ring (stretching vibrations)), 2926 (aliphatic C–H asymmetric stretching vibrations), 2865 cm^−1^ (aliphatic C–H symmetric stretching vibrations), 1610 and 1510 cm^−1^ (aromatic –C=C– stretching vibration), 1240 and 1036 cm^−1^ (aromatic ether C–O–C asymmetric and symmetric stretching vibrations, respectively). The EP containing phosphorus and boron additives (EP1-EP5) exhibited characteristic absorption peaks at 3340 (O–H and N–H stretching vibrations), 2960 and 2865 cm^−1^ (aliphatic C–H stretching vibrations), 1607 and 1510 cm^−1^ (aromatic C=C stretching vibrations), 933 cm^−1^ (aromatic P–O–C stretching vibrations), 754 cm^−1^ (deformation vibrations caused by the 1,2-disubstituted aromatic phosphaphenanthrene rings) and 699 cm^−1^ (deformation vibrations of the aromatic rings).

**Figure 6:**
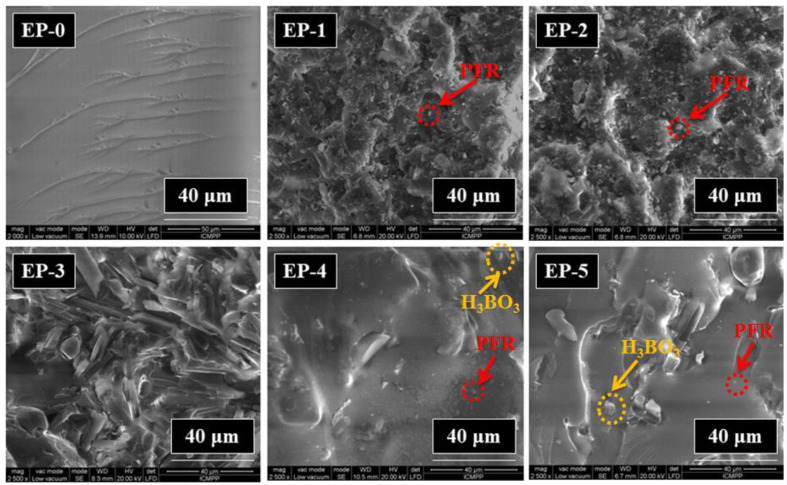
SEM micrographs of epoxy composite surface.

Scanning electron microscopy (SEM) characterization was used to investigate the morphology in the fracture surfaces of cured epoxy resin and its composites. Certain amounts of each sample were immersed under liquid nitrogen, then they were broken by applying mechanical stress. The SEM micrographs taken upon the fractured area of each sample are shown in Figure 6. The neat epoxy resin EP-0 had a smooth fracture surface, and showed a typical brittle fracture. When PFR was incorporated into epoxy resin, different rough fracture surfaces were observed (EP-1, EP-2 and EP-3). The maximum in fracture roughness increased with the amount of PFR content. In the case of EP-4 and EP-5 containing 2 wt% boron and 1.5 and 2 wt% phosphorus, respectively, some agglomerations of H_3_BO_3_ were observed.

### 3.3. Thermal Characterization of Epoxy Composites

**Figure 7:**
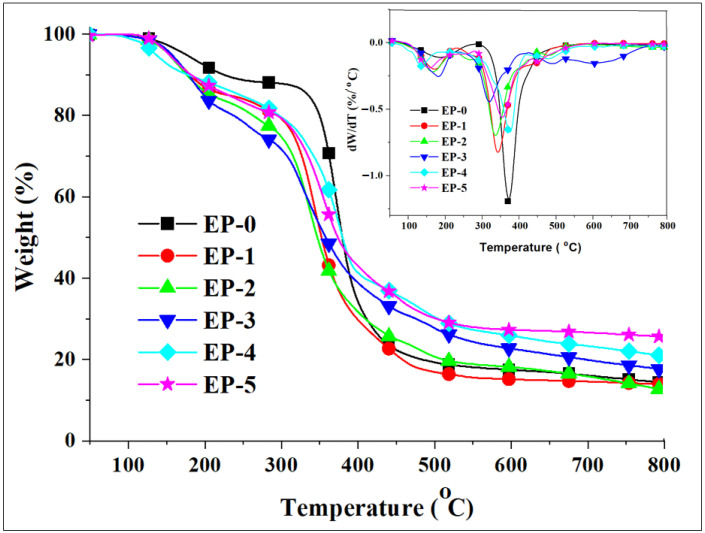
TG and DTG curves (inset) of epoxy composites.

The thermal stability of epoxy composites was evaluated by TGA analysis. The main parameters *T_onset_* (the temperature at which the thermal degradation starts), *T_max_* (the temperature at which the mass loss rate is the highest), and char yield (mass residue remaining after thermal degradation at 800 °C) are summarized in Table 2. Figure 7 shows the TG and DTG curves of epoxy composites. The samples exhibited two steps of weight loss. The first step, in the temperature range of 100–250 °C, was due to the thermal decomposition of PFR, first thermal dehydration of H_3_BO_3_, and second thermal dehydration of the latter [29,44]. The second step of weight loss appeared in the temperature range of 300–550 °C, due to the thermal decomposition of polymer matrix [43]. The *T_onset_* of the second step of thermal decomposition was in the interval 295–342 °C. The introduction of PFR and H_3_BO_3_ slightly decreased *T_onset_* of the samples due to the lower thermal stability of PFR and thermal dehydration process of H_3_BO_3_.

The maximum weight loss temperature in the second step of decomposition was in the interval 330–370 °C. This temperature decreased slightly as the PFR content increased. As can be seen from Figure 7 inset, the neat epoxy thermoset showed the highest weight loss rate (WLR) of 1.161 %/°C at 375 °C. The introduction of phosphorus-containing PFR decreased WLR; for example, for EP-3 containing 4 wt% phosphorus, the WLR was 0.435 %/°C. Moreover, EP-4 and EP-5 containing 2 wt% boron and 1.5 or 2 wt% phosphorus exhibited lower WLR values (0.655 and 0.516 %/°C, respectively).

The char yield values at 800 °C were in the interval 12.9–25.6 wt%. The chars of the samples were relatively stable in the temperature interval of 500–800 °C. The lowest values of the char yield were obtained for the EP-0, EP-1 and EP-2 having lower phosphorus content. An increase in the char yield value was observed for the EP-3 containing 4 wt% phosphorus. The highest values of the char yield were obtained for EP-4 and EP-5 containing 2 wt% boron and 1.5 and 2 wt% phosphorus, respectively. This could be attributed to a synergistic effect of PFR containing phosphorus and H_3_BO_3_ in increasing the char yield values [28]. It is advantageous to have a higher char yield value because in this case a smaller amount of polymeric material is decomposed, thus resulting in a smaller volume of combustible gases that can cause a fire. This effect has been also observed by other researchers when H_3_BO_3_ was used together with a phosphorus-containing compound to improve the flame-resistant characteristics of epoxy resins [38].

**Figure 8:**
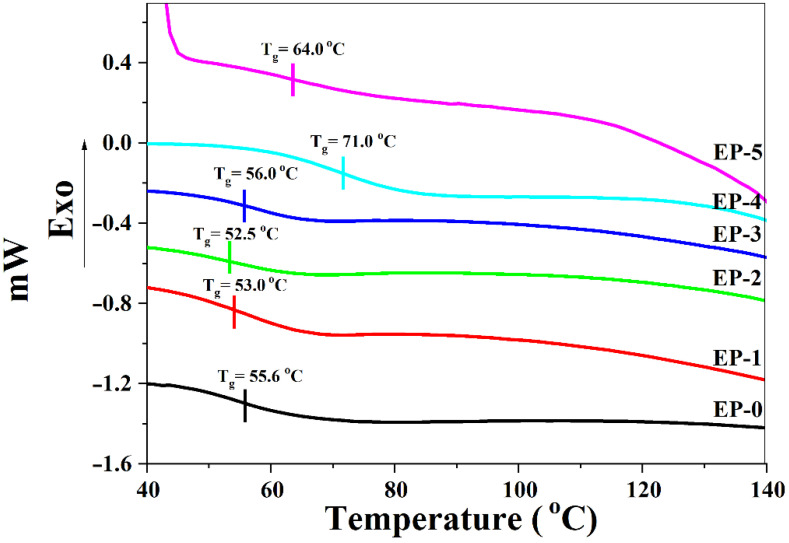
DSC curves of epoxy composites.

The glass transition temperature (*T_g_*) of the samples was in the interval 52.5–71.0 °C (Table 2, Figure 8). An increase in *T_g_* appeared for the samples containing both H_3_BO_3_ and PFR (EP-4 and EP-5), probably because of increased co-interaction between H_3_BO_3_ and PFR with the polymer matrix that resulted in an increase in the rigidity of the macromolecular chains thus increasing the *T_g_* values.

**Figure 9:**
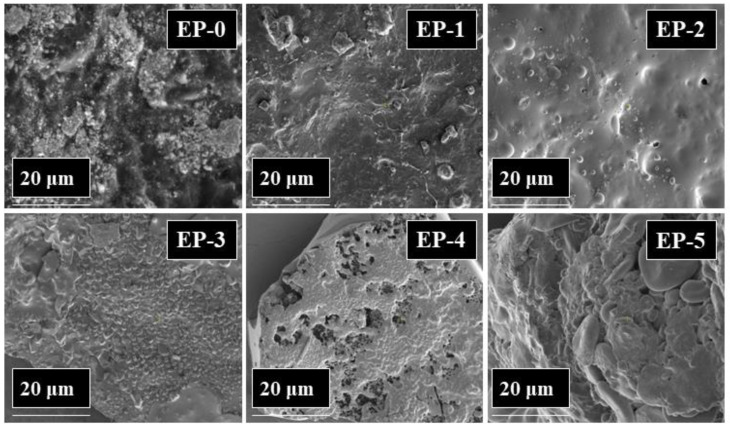
SEM micrographs of epoxy composites heated up to 800 °C, with the heating rate of 10 °C/min, under nitrogen atmosphere.

**Figure 10:**
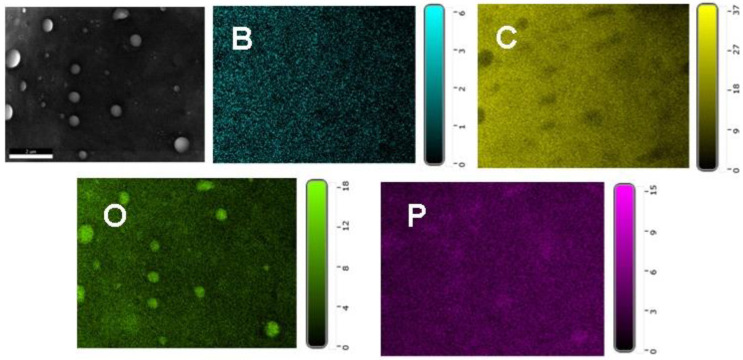
EDX mapping of EP-5 char.

The char residue formed during a fire behaves as a protective layer against the transport of heat and combustible gases. The effectiveness of this protection depends on the quantity and morphology of the char. The SEM images of pyrolyzed composite chars obtained by heating the samples up to 800 °C with the heating rate of 10 °C/min in nitrogen atmosphere are shown in Figure 9. As can be seen from Figure 9, the EP-0 char is porous, having many holes, while the chars of samples containing phosphorus EP-1, EP-2 and EP-3 were more compact and denser. Thus, PFR, a compound derived from DOPO, improved the morphology of the chars. The chars of EP-4 and EP-5 were also compact and dense when compared with those of EP-0. A mapping technique was used to investigate the atom distribution on the char surface. From Figure 10, which presents the EDX mapping of EP-5 char, it can be observed that the phosphorus and boron atoms have been present and are uniformly distributed on the char residue surface.

**Figure 11:**
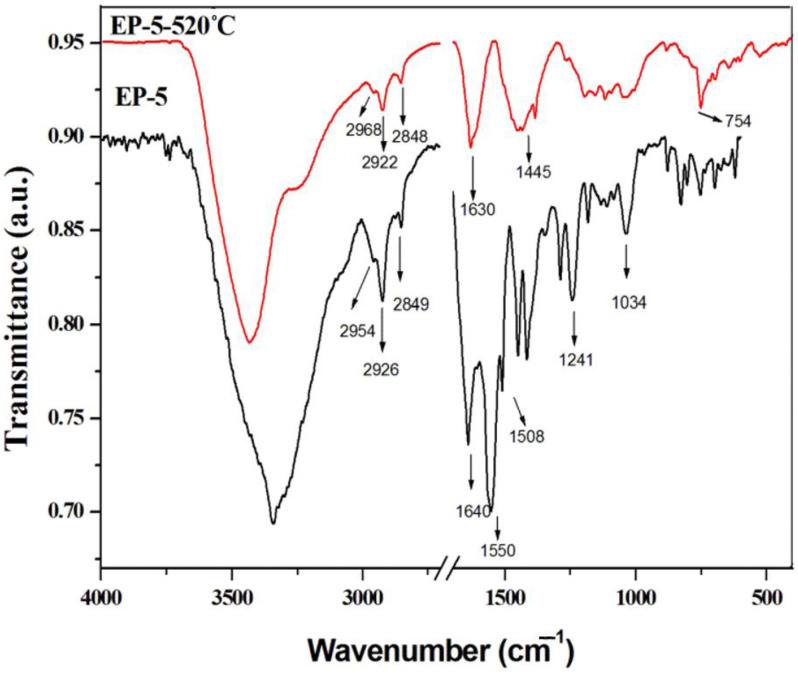
FTIR spectra of EP-5 and EP-5 heated up to 520 °C.

Figure 11 presents the FTIR spectra of EP-5, and EP-5 heated up to 520 °C with the heating rate of 10 °C/min in a nitrogen atmosphere. In the FTIR spectrum of thermally treated EP-5, a decrease in the intensity of characteristic peaks for aliphatic groups at 2968, 2922 and 2868 cm^−1^ can be observed. A strong absorption band characteristic for the presence of aromatic systems appeared at 1630 cm^−1^. The absorption bands at 1440 and 754 cm^−1^ were attributed to P-C and B-O-B bonds in char residue. As was also evidenced by EDX mapping of EP-5 char, it can be concluded that at high temperature the decomposition of EP-5 took place with the degradation of aliphatic groups of epoxy resin and an increase in phosphorus and boron content in the char residue.

### 3.4. Microscale Combustion Calorimetry (MCC) Tests

**Figure 12:**
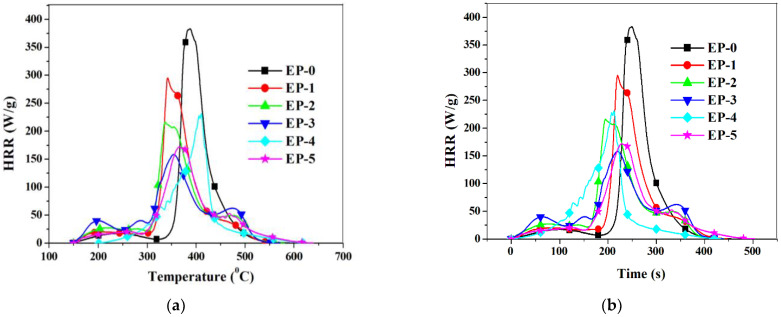
(**a**) Heat release rates versus temperature for epoxy composites; (**b**) Heat release rates versus time for epoxy composites.

Microscale combustion calorimetry (MCC) tests were used to evaluate the flammability of the samples. MCC is an important method for evaluating the combustion behavior of polymer materials [45,46]. The main thermal combustion parameters such as char yield (CY), total heat release, heat release capacity (HRC), the peak of heat release rate (PHRR), the temperature of the heat release peak, and the time to obtain the heat release peak are presented in Table 3. Figure 12a,b presents the dependence of the heat release rate (HRR) with the temperature and time, respectively. As can be seen from Figure 12a, the HRR of EP-0 strongly increased in the interval 340–500 °C. The thermo-oxidative degradation process occurs in several stages with a heat release in each of them. The greatest amount of heat is produced in different stages, dependent of the sample composition. EP-0 exhibited the maximum value of PHRR (383 W/g). A decrease in this parameter appeared by introducing PFR containing phosphorus. Thus, EP-1, EP-2 and EP-3 containing 2, 3 or 4 wt% phosphorus had PHRR values of 295, 216 and 158 W/g, respectively. Moreover, the incorporation of H_3_BO_3_ substantially decreased the PHRR values. Thus EP-5, containing 2 wt% phosphorus and 2 wt% boron, revealed a PHRR value of 172 W/g (Table 3). It can be noticed that a decrease in the time to obtain the heat release peak is caused by increasing the PFR content and the presence of H_3_BO_3_.

EP-0 exhibited the highest value of THR (26.5 kJ/g). A decrease in THR values can be observed in the case of epoxy thermosets containing phosphorus EP-1, EP-2 and EP-3. The lowest value of THR was obtained for EP-3 containing 4 wt% phosphorus (21.9 kJ/mol). The incorporation of H_3_BO_3_ together with compound PFR, in the case of EP-4 and EP-5, substantially decreased THR values, the lowest values being obtained for EP-4 containing 1.5 wt% P and 2 wt% boron (THR = 18.9 kJ/g).

The HRC can be calculated from the MCC test and can be used to classify the flammability of materials, where a low HRC value indicates low flammability of the sample. The HRC of EP-0 was 513 J/(g·K). A substantial decrease in HRC was obtained by incorporating PFR. Thus, sample EP-3 containing 4 wt% phosphorus had an HRC value of 287 J/(g·K). The lowest value of HRC was obtained in the case of EP-5 containing 2 wt% phosphorus and 2 wt% boron (HRC = 213 J/(g·K); this value is lower than those of the EP-2 and EP-3 containing 3 and 4 wt% phosphorus, respectively. It can be concluded that simultaneous incorporation of PFR and H_3_BO_3_ improved considerably the flame resistance of epoxy thermosets.

The char yield (CY) resulting from MCC analysis was in the interval 5.94–20.08 wt%. As it can be seen from Table 3, in the case of EP-1, EP-2 and EP-3, CY values did not increase by increasing the phosphorus content. A substantial increase in CY values were noticed for the samples containing both phosphorus and boron elements Thus, the neat EP-0 exhibited a CY value of 6.27 wt%, while EP-4 and EP-5 had CY values of 18.17 and 20.08 wt%, respectively. The increase in the residue in the case of EP-4 and EP-5 compared with the neat epoxy thermoset EP-0 indicates the formation of a lower amount of combustible gaseous products. Moreover, as was shown in SEM analyses of EP-4 and EP-5 pyrolysis, the char residues exhibited a compact and dense morphology which explains the much lower values for HRC, THR and PHRR determined by MCC tests on compared with those of the neat epoxy thermoset.

## 4. Conclusions

New environmentally friendly flame-resistant epoxy composites were prepared by the incorporation of PFR and H_3_BO_3_ into the epoxy resin. Pyrolysis of epoxy composites containing phosphorus and boron atoms produced a char residue with a compact structure that could act as a barrier to inhibit gaseous products and to insulate the polymer from heat and air. The higher char yield of the samples containing H_3_BO_3_ was beneficial for improving the flame retardance. The SEM micrograph of EP-4 and EP-5 char residues showed the presence of phosphorus and boron atoms distributed relatively uniformly on the char surface. The MCC analyzer revealed that the introduction of 2 wt% boron and 2 wt% phosphorus considerably decreased the flammability of epoxy thermoset. For this sample the THR and HRC greatly decreased, whereas the residue amount increased considerably. In comparison to pure EP-0, the MCC results displayed that the PHRR, THR and HRC values were decreased by 55.03, 22.93 and 58.365%, respectively. In conclusion, the flame resistance of epoxy resin was significantly improved by simultaneous incorporation of a DOPO derivative and H_3_BO_3_. It was demonstrated that these two components did possess a positive role in the condensed phase. In respect of environmental protection, the use of PFR and H_3_BO_3_ as flame retardants for epoxy resins is promising.

## Figures and Tables

**Table 1:** Preparation details of epoxy resin composites.

Sample	Epoxy Resin (EP) (g)	Hardener (HA) (g)	Flame Retardant (PFR) (g)/(%)	H_3_BO_3_ (g)/(%)	Phosphorus (wt%)	Boron (wt%)
EP-0	10	5	-	-	0	0
EP-1	3.96	1.98	1.56/0.12	-	2	0
EP-2	2.35	1.17	1.58/0.08	-	3	0
EP-3	2.92	1.46	3.14/0.23	-	4	0
EP-4	2.48	1.24	0.73/0.04	0.42/0.02	1.5	2
EP-5	3.39	1.70	1.55/0.12	0.86/0.06	2	2

**Table 2:** Thermal properties of epoxy composites.

Sample	T_g_ ^1^ (°C)	T_onset_ ^2^ (°C)	T_max_ ^3^ (°C)	Char Yield ^4^ (wt%)
EP-0	55.6	342	373	14.57
EP-1	53.0	307	344	14.04
EP-2	52.5	303	336	12.91
EP-3	56.0	295	330	17.95
EP-4	71.0	323	373	21.51
EP-5	64.5	308	357	25.60

^1^ Glass transition temperature; ^2^ Temperature at which the thermal degradation starts, in the second step of weight loss; ^3^ Temperature at which the mass loss rate is the highest in the second step of weight loss; ^4^ Char yield after thermal degradation at 800 °C.

**Table 3:** Data obtained by MCC analysis for epoxy composites.

Sample	Char yield (wt%)	THR ^1^ (kJ/g)	HRC ^2^ (J/g*K)	PHRR ^3^ (W/g)	T_PHRR_ ^4^ (°C)	Time ^5^ (s)
EP-0	6.27	26.5	513	383	387	249
EP-1	7.04	24.2	344	295	341	219
EP-2	7.12	23.2	282	215	337	195
EP-3	5.94	21.9	287	158	354	220
EP-4	18.17	18.9	282	231	410	212
EP-5	20.08	20.4	213	172	365	228

^1^ Total heat release; ^2^ Heat release capacity; ^3^ Heat release peak; ^4^ Temperature of heat release peak; ^5^ The time to attain the heat release peak.

## Data Availability

The data that support the findings of the current study are listed within the article.
